# Global Positioning System‐Derived Metrics and Machine Learning Models for Injury Prediction in Professional Rugby Union Players

**DOI:** 10.1002/ejsc.70057

**Published:** 2025-09-24

**Authors:** Xiangyu Ren, Simon Boisbluche, Kilian Philippe, Mathieu Demy, Sami Äyrämö, Ilkka Rautiainen, Shuzhe Ding, Jacques Prioux

**Affiliations:** ^1^ Key Laboratory of Adolescent Health Assessment and Exercise Intervention of Ministry of Education Sino‐French Joint Research Center of Sport Science College of Physical Education and Health East China Normal University Shanghai China; ^2^ Movement, Sport, Health Laboratory University of Rennes 2 Bruz France; ^3^ Department of Sports Sciences and Physical Education École normale supérieure de Rennes Bruz France; ^4^ Rugby Club Vannes French Rugby Federation Vannes France; ^5^ Laboratory of Movement, Balance, Performance and Health (MEPS, EA‐4445) University of Pau and Pays de l’Adour Tarbes France; ^6^ Faculty of Information Technology University of Jyväskylä Jyväskylä Finland; ^7^ Wellbeing Services County of Central Finland Jyväskylä Finland

**Keywords:** analysis, data, injury & prevention, modeling, team sport

## Abstract

In sports, injury prevention is a key factor for success. Although injuries are challenging to predict, new technologies and the application of data science can provide valuable insights. This study aimed to predict injury risk among professional rugby union players using machine learning (ML) models. We analyzed data from 63 professional rugby union players during three seasons, categorized them into forwards and backs, and further classified them into five specific positions (tight five, back row, scrum‐half, inside backs, outside backs). The dataset included GPS data and derived metrics such as total workload in the 1, 2, and 3 weeks prior to injury, acute‐to‐chronic workload ratio over different time windows, monotony, and strain. Injury prediction was assessed separately for different player positions using five ML classification models: logistic regression, naïve Bayes (NB), support vector machine, random forest (RF), and eXtreme gradient boosting (XGBoost). RF performed best for forwards overall, with XGBoost excelling in the tight five and SVM in the back row, whereas among backs, RF led for inside backs and NB for outside backs. Additionally, feature importance plots were used to examine the impact of various factors on injury occurrence. In conclusion, our ML‐based approach can effectively predict injuries, with average *F*1 scores up to 0.66 (± 0.14), particularly when applying a combination of GPS‐derived metrics. Additionally, key characteristics indicative of injury for players in various positions have been successfully identified. These findings underscored the potential of ML to enhance injury prediction and inform tailored training strategies for athletes.

## Highlights

- GPS‐derived workload metrics combined with machine learning models can effectively predict injury in professional rugby union players.
- Injury factors differ by position with forwards influenced by sprint running monotony and backs associated with high‐speed running and acceleration patterns.
- Position‐specific training and workload management strategies can help mitigate injury and enhance athletic performance.

## 1 Introduction

Rugby union, a popular international collision sport, is characterized by high physical intensity and frequent injuries, underscoring the importance of injury prevention and workload management. It presents a multitude of challenges in its matches (Deutsch et al. 2007). These high intensity efforts are also interspersed by low‐intensity activities such as walking and jogging (Cahill et al. 2013; Deutsch et al. 2007). Workload refers to the cumulative stress placed on an athlete over time through training and competition, typically measured by external (e.g., distance run) or internal (e.g., heart rate) workload (Bourdon et al. 2017). Proper workload management not only aims to enhance athletic abilities and performance (Foster et al. 1996) but also reduces the risk of fatigue and injury (Cross et al. 2016; Gabbett and Jenkins 2011).

Elite rugby union has a significantly higher incidence and frequency of injuries compared to most team sports, resulting in considerable training absences (Hind et al. 2020; Williams et al. 2013). These injuries negatively impact team performance, as their occurrence is inversely correlated with league points and Eurorugby Club rankings (Williams et al. 2016). Frequent injuries, whether in individual or team sports, can have serious physical, psychological, and financial consequences (Van Eetvelde et al. 2021). Noncontact injuries, that is, those tissue injuries that are not caused by a direct physical collision, can result in an athlete's inability to participate in sport for at least 1 day (Fuller et al. 2007; Rossi et al. 2018). The research study by Gabbett and Jenkins (2011) highlights a strong correlation between workload and injury rates in professional rugby league players, with field workload notably linked to noncontact training injuries. To address these challenges, the identification and modification of controllable injury risk factors, alongside effective monitoring of workload and recovery, are critical. Such measures are essential to prevent injuries, maintain athlete fitness, and support optimal performance (Van Mechelen et al. 1992; Williams et al. 2017).

For decades, researchers and practitioners in sports science have studied the relationship between athlete workload and injury risk through data analysis (Eckard et al. 2018; Jones et al. 2017; Windt and Gabbett 2017). There are a variety of approaches to quantifying external workload (Vanrenterghem et al. 2017). With the development of global positioning system (GPS) technology, wearable devices are able to monitor and provide real‐time data on the workload during training and competition. Among the most widely used GPS‐derived metrics for assessing training workload are monotony, strain, and the acute‐to‐chronic workload ratio (ACWR) (Oliveira et al. 2021).

ACWR is an indicator used to evaluate the relationship between an athlete's current workload (acute) and long‐term workload (accumulated chronic workload) readiness. Initially, this metric used 1 week of data to represent the acute workload (the “fatigue” state), whereas the rolling average of 4 weeks of data represented the chronic workload (the “fitness” state) (Hulin et al. 2014). Over time, the timeframe ranges for acute to chronic have been adjusted, expanding to vary from 3–14 days and 2–8 weeks, respectively (Andrade et al. 2020; Stares et al. 2018). The calculation method has also evolved to an uncoupled form, where the acute workload component is excluded from the chronic workload calculation (Gabbett et al. 2019). Additionally, the introduction of an exponentially weighted moving average (EWMA) to calculate the acute to chronic workload ratio provides more accurate reflection of current workload by assigning greater weight to recent data (Murray et al. 2016).

Research studies have shown that these metrics can effectively estimate injury risk (Bourdon et al. 2017; Oliveira et al. 2021). For instance, a systematic review by Andrade et al. (2020) found that 90% of the results linked high ACWR with injury risk, particularly when ACWR > 2.0, with a significant increase in the risk of injury, although such values do not always lead directly to injuries. Foster (1998) showed that noncontact injuries were associated with monotony and stress when internal workload was used as the metric. Cummins et al. (2019) used GPS metrics and identified that 2 weeks of higher total distance combined with elevated strain (> 100,000 AU), significantly increased injury risk in the following week. Similarly, covering distances at speeds exceeding 25 km·h^−1^ over 2–3 weeks was associated with a heightened risk of injury.

Advancements in technology and data processing capabilities have led to the widespread adoption of machine learning (ML) in sports science. As a significant branch of artificial intelligence, ML algorithms are able to automatically learn and extract valuable information from collected sports data, which in turn plays an important role in game analysis, tactical development, performance evaluation and outcome prediction (Naglah et al. 2018). The suitability of tree‐based machine learning models for injury risk prediction in sports has been supported by systematic reviews highlighting their ease of visualization and interpretation (Leckey et al. 2025; Van Eetvelde et al. 2021). These models can be enhanced through integrated methods such as boosting and bagging or adapted to account for cost‐sensitive factors.

The goal of this study was to evaluate the predictive performance of five ML models, including tree‐based and alternative approaches, to determine the variables most significantly influencing injury prediction. By identifying the most effective models and key predictors, this research study aims to advance injury prevention and management strategies in rugby union. We hypothesize that metrics such as ACWR will have a stronger predictive value for injury risk than other workload metrics and that identifying the most effective ML models and predictors will help advance injury prevention and management strategies in professional rugby union players.

## 2 Methods

### 2.1 Experimental Approach to the Problem

**FIGURE 1:**
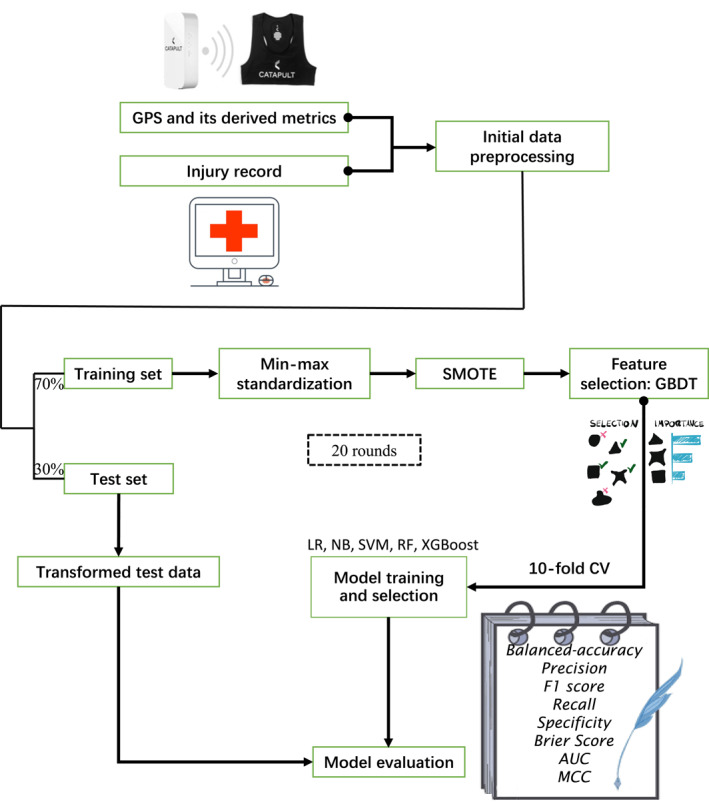
Schematic describing the predictive injury model for professional rugby union players.

This study aimed to examine the impact of GPS‐derived workload metrics on time‐loss injury prediction using data collected over three rugby seasons (2021–2024). To address multicollinearity, we removed metrics with a variance inflation factor > 10 and pairwise correlations above 0.9, retaining 17 workload‐related GPS metrics as the basis for subsequent feature derivation (Akinwande et al. 2015). Injury data, recorded by the team staff, included details such as injury date, cause, location, time of absence, and type of injury. Due to the incidental nature of contact injuries, only noncontact injuries were addressed in this study, totaling 71 cases. The schematic overview of the study design is presented in Figure 1.

### 2.2 Subject

The study involved 63 male professional rugby union players (age: 25.7 ± 5.1 years; height: 190.0 ± 10.0 cm; weight: 103.4 ± 15.8 kg) from a team in the French Pro D2 second division rugby championship. Players were grouped into forwards and backs, with further sub‐categorization into five playing positions: tight five (props, hooker, locks), back row (flankers, number 8), scrum‐half, inside backs (fly‐half, centers), and outside backs (wings, full‐back) (Cahill et al. 2013). All players were informed of the monitoring procedures. Prior to signing an informed consent form following the Declaration of Helsinki, players were aware of the research benefits and agreed to share daily training data. The study followed the ethical guidelines of the University of Rennes and its associated research laboratories.

### 2.3 Procedures

#### 2.3.1 Workload Monitoring

External workload was assessed using a GPS device (Vector X7 sensor, Catapult Innovations, Australia) equipped with integrated 10 Hz GPS, 100 Hz triaxial accelerometer, gyroscope, and 100 Hz magnetometer. The device was activated and placed in an open area half an hour before training to ensure a reliable satellite connection could be established. Each player wore a specialized vest containing sensors (81 × 43 × 16 mm, weighing 53 g) positioned between the shoulder blades on the upper back. Previous studies have validated the effectiveness of this device in reliably monitoring running and acceleration metrics in team sports (Clavel et al. 2022; Cousins et al. 2023). Specialized GPS software (Openfield Console 3.7) was used to export GPS and inertial data, which were then stored in OpenField Cloud for subsequent analysis.

#### 2.3.2 Calculations of Metrics

Based on GPS metrics, this study calculated the cumulative external workload for the injury week (W1), the 2 weeks preceding the injury (W2), and the 3 weeks prior to the injury (W3). Additionally, metrics such as EWMA ACWR, monotony, and strain were computed. Four different ratios for calculating EWMA ACWR were used, specifically set at 3:14, 3:21, 7:21, and 7:28 for the ratio of acute to chronic days (Maupin et al. 2020; West et al. 2021). EWMA for any given day was calculated using the following formula:

$$\text{EWM}A_{\text{today}}=\text{workloa}d_{\text{today}}\times {ƛ}_{a}+\left[\left(1-{ƛ}_{a}\right)\times \text{EWM}A_{\text{yesterday}}\right].$$

In this formula, ${ƛ}_{a}$ (workload decay rate) was calculated as 2/(N+1), where N is the length of the time window. Its value ranges from 0 to 1. The daily EWMA ACWR value refers to the ratio between the acute EWMA value and the chronic EWMA value calculated using the method described above (Murray et al. 2016).

Monotony, a measure of workload variability, was calculated as the daily mean workload divided by the standard deviation over the week. Strain, representing the overall stress generated by training, was determined as the product of workload and monotony (Foster 1998).

#### 2.3.3 Data Preprocessing

In this study, we utilized workload data captured by 17 GPS metrics on the day of injury and derived metrics from these data, including workloads during the 1, 2, and 3 weeks prior to injury, four types of EWMA ACWR, monotony, and strain. Altogether, these combinations resulted in 10 sets of features, producing a total of 170 input features used for modeling (Table 1). To reduce redundancy, features with Pearson correlation > 0.7 were removed while retaining the original GPS metrics. The number of features retained per positional group was as follows: 23 for forwards, 18 for backs, 31 for the tight five, 25 for the back row, 20 for scrum‐half, 26 for inside backs, and 17 for outside backs.

**TABLE 1:** GPS and its derivative data explanation.

	Feature	Units	Definition
GPS metrics	Total distance (TD)	Meter	Assessed from GPS, corresponds to the total distance covered by players during the ball‐in‐play time of training
	Medium‐speed running distance (MSR)	Meter	Distance covered between 15 and 18 km·h^−1^
	High‐speed running distance (HSR)	Meter	Distance covered between 18 and 21 km·h^−1^
	Very high‐speed running distance (VHSR)	Meter	Distance covered between 21 and 25 km·h^−1^
	Sprint running distance (SR)	Meter	Distance covered above 25 km·h^−1^
	Repeated high‐intensity efforts (RHIE)	Number	Three consecutive high‐intensity efforts (contact. acceleration. or sprint) occurring within 21 s
	Contact involvement total count	Number	The number of contact involvement
	Acceleration zone 1 (AZ1)	Number	The number of accelerations between 2 and 2.5 m·s^−2^
	Acceleration zone 2 (AZ2)	Number	The number of accelerations between 2.5 and 3 m·s^−2^
	Acceleration zone 3 (AZ3)	Number	The number of accelerations above 3 m·s^−2^
	Greater than acceleration zone 1 (gtAZ1)	Number	The number of accelerations above 2 m·s^−2^
	Greater than acceleration zone 2 (gtAZ2)	Number	The number of accelerations above 2.5 m·s^−2^
	gtAZ1 + DZ1	Number	The number of accelerations (AZ1) and deceleration (DZ1) above 2 m·s^−2^
	gtAZ2 + DZ2	Number	The number of accelerations and deceleration above 2.5 m·s^−2^
	Greater than acceleration distance zone 1 (gtAZ1)	Meter	Distance at acceleration of above 2 m·s^−2^
	Greater than deceleration zone 2 (gtDZ2)	Number	The number of decelerations above 2.5 m·s^−2^
	Deceleration zone 3 (DZ3)	Number	The number of decelerations above 3 m·s^−2^
	Today		Workload on the day of injury
GPS derived metrics	EWMA 3:14		Exponentially weighted moving average acute (3‐day) to chronic (14‐day) workload ratio calculated based on the above 17 GPS metrics
	EWMA 3:21		Exponentially weighted moving average acute (3‐day) to chronic (21‐day) workload ratio calculated based on the above 17 GPS metrics
	EWMA 7:21		Exponentially weighted moving average acute (7‐day) to chronic (21‐day) workload ratio calculated based on the above 17 GPS metrics
	EWMA 7:28		Exponentially weighted moving average acute (7‐day) to chronic (28‐day) workload ratio calculated based on the above 17 GPS metrics
	Monotony		Monotony calculated based on the above 17 GPS metrics
	Strain		Strain calculated based on the above 17 GPS metrics
	Cumulative workload for 1 week (Total 1)		Cumulative workload for 1 week calculated based on the above 17 GPS metrics
	Cumulative workload for 2 weeks (Total 2)		Cumulative workload for 2 weeks calculated based on the above 17 GPS metrics
	Cumulative workload for 3 weeks (Total 3)		Cumulative workload for 3 weeks calculated based on the above 17 GPS metrics

To optimize model performance, we applied standard preprocessing techniques. The raw dataset initially contained tens of thousands of training and match records across three seasons. After applying inclusion criteria requiring 3 weeks of continuous training data prior to the injury day, including both injured and noninjured players, and excluding nonrelevant conditions such as inflammation or COVID, the ML original dataset comprised 2025 records. During data cleaning, 56.7% of the original dataset records were excluded by removing rows containing missing feature values or extreme values. The final dataset included 877 records, encompassing both injured and noninjured cases. The number of records and corresponding noncontact injuries for each playing position were as follows: 320 and 27 for tight five, 152 and 6 for back row, 107 and 5 for scrum‐half, 157 and 17 for inside backs, and 141 and 14 for outside backs. The dataset was then split into training and testing sets, with 70% of the data allocated for training and 30% for testing to evaluate model performance. Given the severe imbalance in the data, we employed the synthetic minority over‐sampling technique (SMOTE) to balance the positive and negative samples in the training set by generating new minority class samples through interpolation. For feature selection, we used gradient boosting decision trees to screen features, retaining those with an important score > 0.01.

#### 2.3.4 Model Development

Because the dataset's output labels were categorized as injured/uninjured, the problem was framed as a binary classification task. In this study, we used the Python 3.12 programming environment and several classifiers from the scikit‐learn library (version 1.4.2) to model the data. We employed 10‐fold cross‐validation to optimize hyperparameters, including logistic regression (LR), naïve Bayes (NB), support vector machine (SVM), random forest (RF), and eXtreme gradient boosting (XGBoost) (see Supporting Information S1 for detailed principles and formulas). To access the randomness associated with data splitting, the entire process was repeated 20 times, and the average of the evaluation metrics was calculated to ensure robustness and reproducibility (Song et al. 2021). The parameters of the classification models are shown in Table 2.

**TABLE 2:** Machine learning models and parameters.

Model	Parameter	Meaning	Value
Logistic regression	C	The C parameter controls the inverse strength of regularization, with smaller values leading to stronger regularization and larger values to weaker regularization	[0.01, 0.1, 1, 10, 100]
	max_iter	The maximum number of iterations, used to control the convergence of the solver	2000
	solver	The algorithm used to optimize the model's cost function during training	'lbfgs', 'liblinear'
Naïve Bayes	priors	Prior probabilities for the classes	None
	var_smoothing	Variance added to each feature to account for stability	1e‐9
Support vector machine	C	A parameter that balances model simplicity and training error	[0.1, 1, 10, 100]
	kernel	The algorithm used to optimize the model's cost function during training	'linear', 'rbf'
Random forest	n_estimators	Number of weak learners (trees)	[50, 100]
	max_depth	Maximum allowed depth for trees	[1, 3, 5, 7, 9, 11, 13, 15, 17, 19]
	min_samples_split	Number of samples required to split an internal node	[2, 4, 6]
	min_samples_leaf	Minimum number of samples required for leaf nodes	[1, 2, 4]
eXtreme gradient boosting	n_estimators	Number of weak learners (trees)	[50, 100]
	learning_rate	Reduce the step size of each step to prevent overfitting	[0.01, 0.1, 0.2]
	max_depth	Maximum allowed depth for trees	[3, 5, 7, 9]
	subsample	The proportion of data used in each tree training to the entire training set	[0.6, 0.8, 1.0]

### 2.4 Statistical Analysis

#### 2.4.1 Performance Evaluations

In this study, multiple performance metrics were employed to comprehensively evaluate the modeling results. These metrics included balanced accuracy, precision, recall (sensitivity), *F*1‐score, specificity, Brier score, Matthews correlation coefficient (MCC), and area under the receiver operating characteristic curve (AUC). Balanced accuracy refers to a metric used to evaluate the performance of a classification model, especially when dealing with imbalanced datasets. Unlike traditional accuracy, which can be misleading in cases where one class dominates the dataset, balanced accuracy adjusts for class imbalance by considering both the true positive rate (sensitivity) and the true negative rate (specificity). Precision measures the ratio of true positive samples among those predicted as positive, indicating how many of the predicted positive cases are genuinely positive. Recall, or sensitivity, represents the proportion of actual positive samples correctly identified by the model, reflecting its ability to detect all positive instances. The *F*1‐score, which is the harmonic mean of precision and recall, provides a single value to assess the balance between these two metrics, with higher scores indicating better overall performance. Specificity measures the proportion of actual negative samples accurately predicted as negative by the model, demonstrating its effectiveness in identifying negative cases. The Brier score evaluates the accuracy of probabilistic predictions by averaging the squared differences between predicted probabilities and actual outcomes, where a lower score signifies better performance. MCC is a metric that measures the quality of binary classification, taking into account all four components of the confusion matrix and providing a balanced evaluation, especially useful for imbalanced datasets.

$$\text{Balanced}\;\text{accuracy}=\frac{1}{2}\left(\text{Recall}+\text{Specificity}\right)=\frac{1}{2}\left(\frac{\text{TP}}{\text{TP}+\text{FN}}+\frac{\text{TN}}{\text{TN}+\text{FP}}\right),$$

where TP is true positives, TN is true negatives, FP is false positives, and FN is false negatives.

$$\text{Precision}=\frac{\text{TP}}{\text{TP}+\text{FP}},$$

where TP is true positives and FP is false positives.

$$\text{Recall}=\frac{\text{TP}}{\text{TP}+\text{FN}},$$

where TP is true positives and FN is false negatives.

$$F1\;\text{score}=2\times \frac{\text{Precision}\times \text{Recall}}{\text{Precision}+\text{Recall}}=\frac{2\times \text{TP}}{2\times \text{TP}+\text{FP}+\text{FN}},$$

where TP is true positives, FP is false positives and FN is false negatives.

$$\text{Specificity}=\frac{\text{TN}}{\text{TN}+\text{FP}},$$

where TN is true negatives and FP is false positives.

$$\text{Brier}\;\text{score}=\frac{1}{N}\sum_{i=1}^{N}\sum {\left(p_{i}-o_{i}\right)}^{2},$$

where N is the total number of samples, $p_{i}$ is the predicted probability for the ith sample, $o_{i}$ is the actual outcome for the ith sample (0 or 1).

$$\text{MCC}=\frac{\text{TP}\times \text{TN}-\text{FP}\times \text{FN}}{\sqrt{\left(\text{TP}+\text{FP}\right)\left(\text{TP}+\text{FN}\right)\left(\text{TN}+\text{FP}\right)\left(\text{TN}+\text{FN}\right)}},$$

where TP is true positives, TN is true negatives, FP is false positives and FN is false negatives.

The ROC curve plots the true positive rate (TPR) against the false positive rate (FPR). A higher AUC value indicates superior model performance, as it reflects the model's ability to effectively distinguish between positive and negative samples.

$$\text{TPR}=\frac{\text{TP}}{\text{TP}+\text{FN}},$$

$$\text{FPR}=\frac{\text{FP}}{\text{FP}+\text{TN}},$$

where TP is true positives, TN is true negatives, FP is false positives and FN is false negatives.

#### 2.4.2 Feature Importance

During each training process, the selected features were evaluated based on the best‐performing model on the test set using permutation importance. To improve the robustness of feature importance evaluation across multiple runs, the study combined the average permutation importance score of each feature with its selection frequency (the proportion of runs in which the feature was selected). The final weighted feature importance (WFI) was obtained by multiplying the average permutation importance score by the selection frequency. Based on WFI, the top 10 features were identified, and their average raw permutation importance scores were reported for further analysis.

## 3 Results

**FIGURE 2:**
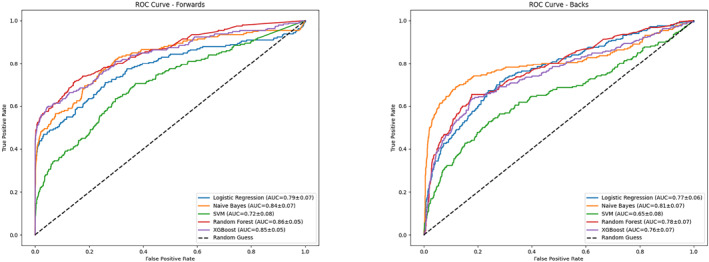
Area under the curve of five classifiers for forwards and backs.

**FIGURE 3:**
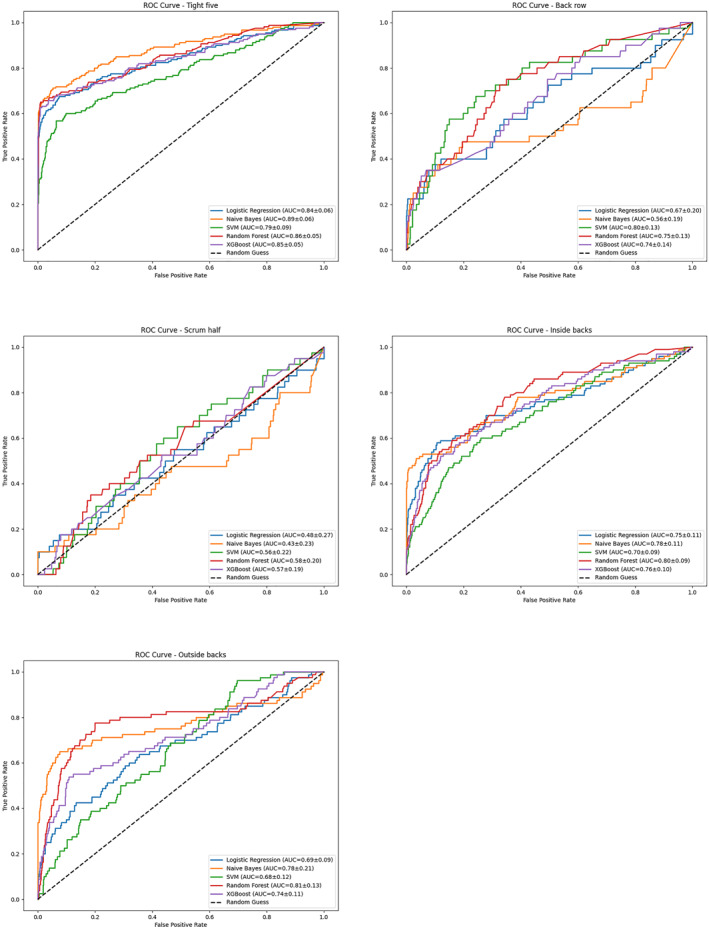
Area under the curve of five classifiers for players in five positions.

Table 3 compares the evaluation metrics scores of different models in each position. Among the forwards, RF achieved the highest balanced accuracy (0.76), precision (0.74), *F*1 (0.61), MCC (0.60), and AUC (0.86) (Figure 2), whereas NB performed best among the backs. When positions were further divided, XGBoost attained the highest precision (0.87) and *F*1 (0.66) for the tight five, whereas NB achieved the best AUC (0.89) (Figure 3). In the back row, RF had highest balanced accuracy (0.63) and recall (0.30), but SVM outperformed in AUC (0.80). All models performed poorly in the scrum‐half position. For inside backs, RF achieved the highest accuracy (0.45), *F*1 (0.43), and MCC (0.38), with an AUC of 0.82. For outside backs, NB led in balanced accuracy (0.78), recall (0.69), and *F*1 (0.65), whereas RF achieved the highest AUC (0.81).

**TABLE 3:** Comparison of five machine learning models (mean value ± standard deviation).

Position	Model	Balanced accuracy	Precision	Recall	*F*1 score	Specificity	Brier score	Matthews correlation coefficient
Forwards	Logistic regression	0.71 ± 0.06	0.23 ± 0.06	0.56 ± 0.14	0.32 ± 0.06	0.86 ± 0.04	0.11 ± 0.02	0.28 ± 0.08
	Naive Bayes	0.74 ± 0.06	0.27 ± 0.09	0.61 ± 0.12	0.37 ± 0.09	0.86 ± 0.06	0.12 ± 0.05	0.33 ± 0.10
	Support vector machine	0.61 ± 0.07	0.37 ± 0.20	0.26 ± 0.14	0.29 ± 0.14	0.97 ± 0.02	0.07 ± 0.01	0.26 ± 0.15
	Random forest	0.76 ± 0.07	0.74 ± 0.16	0.55 ± 0.15	0.61 ± 0.11	0.98 ± 0.01	0.05 ± 0.01	0.60 ± 0.11
	eXtreme gradient boosting	0.76 ± 0.07	0.61 ± 0.14	0.56 ± 0.14	0.56 ± 0.10	0.97 ± 0.02	0.05 ± 0.01	0.54 ± 0.10
Backs	Logistic regression	0.71 ± 0.05	0.23 ± 0.03	0.63 ± 0.12	0.33 ± 0.04	0.78 ± 0.04	0.16 ± 0.02	0.27 ± 0.06
	Naive Bayes	0.78 ± 0.07	0.41 ± 0.11	0.68 ± 0.13	0.50 ± 0.12	0.89 ± 0.05	0.11 ± 0.04	0.46 ± 0.13
	Support vector machine	0.59 ± 0.07	0.32 ± 0.19	0.22 ± 0.13	0.26 ± 0.15	0.95 ± 0.02	0.10 ± 0.02	0.20 ± 0.16
	Random forest	0.69 ± 0.08	0.43 ± 0.13	0.43 ± 0.17	0.42 ± 0.13	0.94 ± 0.02	0.09 ± 0.01	0.37 ± 0.14
	eXtreme gradient boosting	0.69 ± 0.06	0.39 ± 0.10	0.45 ± 0.13	0.41 ± 0.09	0.92 ± 0.03	0.10 ± 0.02	0.35 ± 0.10
Tight five	Logistic regression	0.78 ± 0.08	0.35 ± 0.10	0.69 ± 0.15	0.46 ± 0.10	0.88 ± 0.04	0.10 ± 0.02	0.42 ± 0.12
	Naive Bayes	0.80 ± 0.07	0.48 ± 0.15	0.68 ± 0.15	0.54 ± 0.10	0.92 ± 0.04	0.10 ± 0.02	0.51 ± 0.11
	Support vector machine	0.79 ± 0.07	0.59 ± 0.22	0.63 ± 0.15	0.58 ± 0.12	0.95 ± 0.04	0.10 ± 0.02	0.56 ± 0.14
	Random forest	0.79 ± 0.07	0.73 ± 0.20	0.60 ± 0.14	0.64 ± 0.10	0.97 ± 0.03	0.10 ± 0.02	0.62 ± 0.12
	eXtreme gradient boosting	0.77 ± 0.08	0.87 ± 0.17	0.56 ± 0.16	0.66 ± 0.14	0.99 ± 0.02	0.10 ± 0.02	0.67 ± 0.14
Back row	Logistic regression	0.63 ± 0.14	0.15 ± 0.14	0.35 ± 0.29	0.21 ± 0.17	0.91 ± 0.06	0.13 ± 0.07	0.18 ± 0.19
	Naive Bayes	0.61 ± 0.13	0.28 ± 0.36	0.28 ± 0.26	0.25 ± 0.26	0.95 ± 0.05	0.07 ± 0.04	0.22 ± 0.28
	Support vector machine	0.59 ± 0.13	0.24 ± 0.36	0.23 ± 0.26	0.21 ± 0.26	0.95 ± 0.04	0.06 ± 0.03	0.19 ± 0.29
	Random forest	0.63 ± 0.12	0.28 ± 0.35	0.30 ± 0.25	0.25 ± 0.25	0.95 ± 0.07	0.07 ± 0.03	0.24 ± 0.27
	eXtreme gradient boosting	0.60 ± 0.13	0.20 ± 0.27	0.25 ± 0.26	0.21 ± 0.23	0.96 ± 0.03	0.08 ± 0.04	0.18 ± 0.24
Scrum‐half	Logistic regression	0.53 ± 0.13	0.11 ± 0.17	0.20 ± 0.25	0.13 ± 0.18	0.86 ± 0.08	0.14 ± 0.05	0.05 ± 0.21
	Naive Bayes	0.52 ± 0.11	0.12 ± 0.27	0.10 ± 0.21	0.10 ± 0.22	0.94 ± 0.06	0.09 ± 0.04	0.05 ± 0.24
	Support vector machine	0.48 ± 0.07	0.05 ± 0.22	0.03 ± 0.11	0.03 ± 0.15	0.93 ± 0.06	0.11 ± 0.05	−0.02 ± 0.17
	Random forest	0.48 ± 0.02	0.00 ± 0.00	0.00 ± 0.00	0.00 ± 0.00	0.95 ± 0.05	0.09 ± 0.03	−0.05 ± 0.04
	eXtreme gradient boosting	0.50 ± 0.09	0.07 ± 0.24	0.05 ± 0.15	0.06 ± 0.18	0.94 ± 0.05	0.11 ± 0.04	0.01 ± 0.20
Inside backs	Logistic regression	0.72 ± 0.10	0.34 ± 0.11	0.59 ± 0.22	0.42 ± 0.11	0.86 ± 0.06	0.13 ± 0.02	0.36 ± 0.14
	Naive Bayes	0.70 ± 0.09	0.34 ± 0.12	0.53 ± 0.20	0.40 ± 0.12	0.87 ± 0.06	0.13 ± 0.04	0.33 ± 0.14
	Support vector machine	0.60 ± 0.09	0.35 ± 0.30	0.26 ± 0.17	0.28 ± 0.19	0.93 ± 0.05	0.11 ± 0.03	0.22 ± 0.22
	Random forest	0.69 ± 0.11	0.45 ± 0.27	0.46 ± 0.23	0.43 ± 0.20	0.92 ± 0.05	0.10 ± 0.02	0.38 ± 0.23
	eXtreme gradient boosting	0.69 ± 0.10	0.42 ± 0.18	0.46 ± 0.20	0.42 ± 0.16	0.92 ± 0.04	0.11 ± 0.03	0.36 ± 0.18
Outside backs	Logistic regression	0.62 ± 0.07	0.19 ± 0.06	0.42 ± 0.18	0.26 ± 0.08	0.82 ± 0.07	0.16 ± 0.03	0.17 ± 0.10
	Naive Bayes	0.77 ± 0.14	0.41 ± 0.17	0.65 ± 0.27	0.49 ± 0.19	0.90 ± 0.05	0.11 ± 0.04	0.45 ± 0.22
	Support vector machine	0.55 ± 0.09	0.23 ± 0.26	0.17 ± 0.16	0.19 ± 0.19	0.93 ± 0.04	0.12 ± 0.03	0.13 ± 0.21
	Random forest	0.71 ± 0.12	0.46 ± 0.27	0.50 ± 0.24	0.45 ± 0.21	0.93 ± 0.06	0.09 ± 0.03	0.41 ± 0.23
	eXtreme gradient boosting	0.68 ± 0.13	0.34 ± 0.25	0.45 ± 0.26	0.37 ± 0.22	0.90 ± 0.06	0.11 ± 0.04	0.31 ± 0.24

**FIGURE 4:**
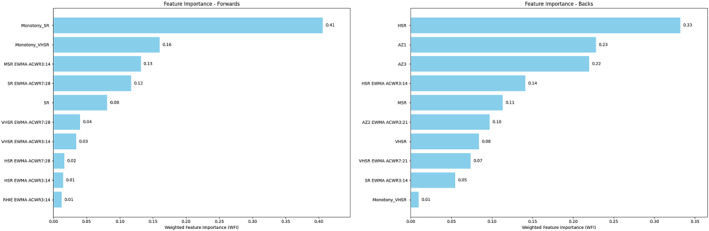
Feature importance plots for forwards and backs.

**FIGURE 5:**
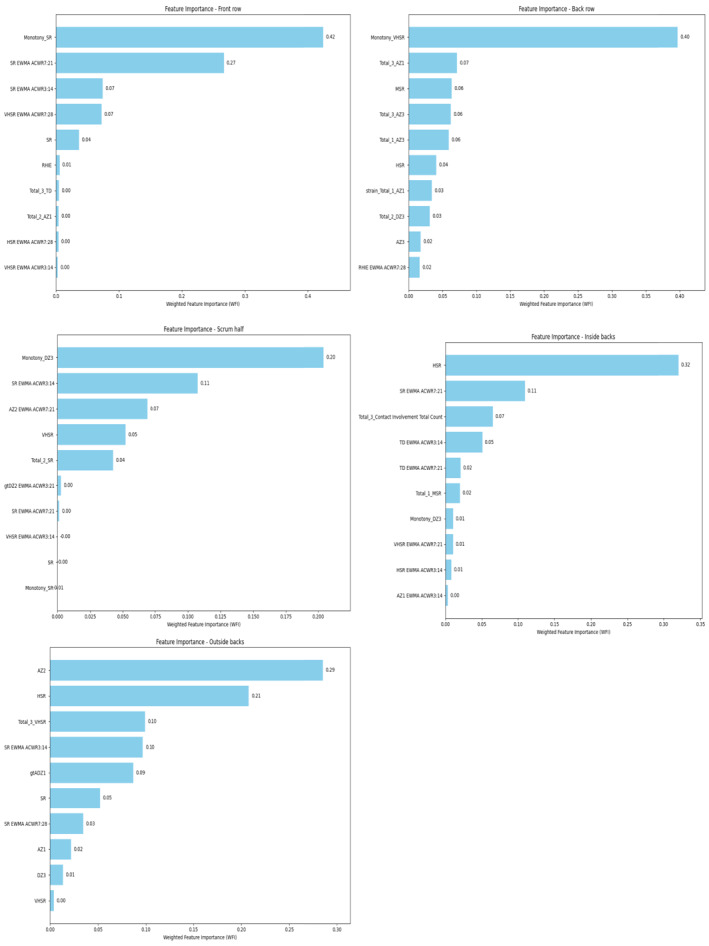
Feature importance plots for players in five positions.

The feature importance analysis for the different playing positions, as shown in Figures 4 and 5, highlights significant differences in the factors contributing to injuries across various positions in rugby union. For the forwards, features such as the monotony of sprint running (SR) and very high‐speed running (VHSR) distance, and EWMA ACWR of medium‐speed running (MSR) distance over a 3:14 window contributed most to the model. In contrast, for the backs, features on the injury day ranked higher in importance, including high‐speed running (HSR) distance, the number of accelerations in the 2–2.5 m·s^−2^ (AZ1) and above 3 m·s^−2^ zone (AZ3).

For the tight five players, the most important features identified were SR monotony, with the EWMA of SR over a 7:21 window contributing significantly. For the back row players, VHSR monotony, 3‐week cumulative AZ1, and MSR ranked highest in feature importance. The scrum‐half position showed a different pattern, with the most critical features being the number of decelerations above 3 m·s^−2^ (DZ3) monotony and EWMA of SR over a 3:14 window. For inside backs, HSR, and SR over a 7:21 window contributing were most important. The number of accelerations in the 2.5–3 m·s^−2^ zone (AZ2) and HSR on the injury day affected the most important feature of outside back injuries.

## 4 Discussion

In the present study, we analyzed the injury risk factors of forwards, backs, and the five specific positions within these categories using data from three seasons of rugby union, including GPS and its derived metrics. This is the first study in this field to apply ML models, including LR, NB, SVM, RF, and XGBoost, to construct injury prediction models. Model performance was evaluated using metrics such as balanced accuracy, precision, recall, *F*1‐score, specificity, Brier score, MCC, and AUC. In addition, we ranked features according to their contribution to injury prediction. The results demonstrated that RF performed best for forwards, with XGBoost excelling in the tight five and SVM in the back row. NB demonstrated the highest performance for backs overall. Within the subgroups, RF outperformed others for inside backs, and NB remained best for outside backs. Additionally, position‐specific performance patterns were evident, reflecting the influence of differing physical demands on injury.

LR and NB models were selected for their straightforward probabilistic classification. SVM known for their ability to handle high‐dimensional data and model complex nonlinear boundaries, were also considered. Tree‐based models are favored for their ease of visualization and interpretation, their ability to effectively model complex nonlinear interactions among multiple predictor variables, and their flexibility to be extended into ensemble methods such as boosting and bagging or adapted to be cost‐sensitive (Naglah et al. 2018). For example, Rossi et al. (2018) developed an injury predictor using features extracted from GPS data, including current and previous external workload, derived ACWR, monotony, and personal characteristics such as age and BMI. Their study found that decision tree was the best‐performing model, capable of predicting 80% of injuries with 50% precision, and an AUC of 0.76 (Rossi et al. 2018). In the systematic review by Van Eetvelde et al. (2021), most of the studies showed that the ML prediction methods performed well in predicting injuries, with AUC values of the predictions ranging between 0.64 and 0.87, accuracies ranging between 75% and 82.9%, sensitivities ranging from 55.6% to 94.5%, specificities ranging from 74.2% to 87%, and precision ranging from 50% to 85%. The precision in this study differed from the results of this review, ranging between 15% and 87% (excluding the scrum‐half position). This can be explained by the fluctuation in precision due to the small amount of data available for the scrum‐half position when the positions were further divided. The performance of the other metrics aligned with the integrated findings of the review. Additionally, following the review's recommendations, we incorporated the Brier score to evaluate the model's ability to predict event occurrences, achieving scores ranging from 0.05 to 0.16 (Van Eetvelde et al. 2021).

Furthermore, injuries to players in specific positions are significantly affected by different features. When the players were divided into two groups, high‐intensity running metrics primarily influenced the forwards, whereas medium‐ and low‐intensity metrics were more impactful for the backs. In particular, tight five players were particularly impacted by repetitive high‐intensity efforts, back row players were influenced by acceleration, highlighting the need for emphasis on explosive power and efficient movement mechanics. Scrum‐halves required endurance at moderate intensities for continuous decision‐making, passing, and tactical play. However, the predictive models developed for this position demonstrated relatively low performance, indicating that the feature importance results for this subgroup should be interpreted with caution. Inside backs were engaged in fast‐paced, explosive movements, and medium‐intensity actions, crucial for both attacking and defending situations. Additionally, acceleration and deceleration were crucial for outside backs, as they often need to quickly start, stop, or change direction to respond to dynamic situations. The findings of this study align with those of Ruddy et al. (2018), who applied supervised learning techniques to predict hamstring injuries in elite Australian football players and identified HSR as the primary mechanism leading to injury. Similarly, outside backs in rugby union experience higher rates of injury‐related absences compared to other backs (Brooks and Kemp 2011), attributed to their faster running speeds and the greater number of sprints required during matches. To mitigate the risk of injury, it is therefore crucial to carefully manage the workload associated with high‐intensity running for these players.

It is well documented that rapid increases in workload and acute increases in ACWR are strongly associated with increases in team injury rates. Therefore, measuring absolute and relative workload in team sports to monitor workload progression and to rationalize training programs continues to be considered best practice (Carey et al. 2018; Murray et al. 2016). However, the use of ACWR has also faced criticism, with some researchers questioning its validity and practical utility due to inconsistencies in its predictive value and methodological limitations (Impellizzeri et al. 2020, 2021). Despite these debates, ACWR remains a widely utilized tool in workload management (Ren et al. 2024; West et al. 2021). In the study by Thornton et al. (2017) on the importance of injury incidence in professional rugby league players, it was noted that for second row players, the 7:28 rolling average of the ACWR for HSR > 5 m·s^−1^ was the most significant factor contributing to injury risk. This is due to the significant impact of frequent collisions during matches and the need for high‐intensity acceleration and deceleration efforts in this position (Thornton et al. 2017). In our study, ACWR calculated over different time windows (e.g., 3:14, 3:21, and 7:28) was consistently identified as an important predictor in the feature importance analysis. This supports our original hypothesis that ACWR plays a meaningful role in injury prediction among professional rugby union players.

A key limitation of this study is its reliance on data from a single professional rugby union team, which restricts the sample size and may impact the reliability and generalizability of the ML models. When attempting to further divide the tight five into front row and second row, the injury prediction accuracy for the second row was almost zero. This is also evident from the poor model performance for scrum‐halves, indicating that 3 years of noncontact injury data is insufficient for reliable predictions. Moreover, although the data analysis and statistical methods used can be directly and immediately applied to other rugby union teams and broader team sports contexts, the applicability of the findings to injury risk associations may vary due to differences between teams. These differences arise primarily from the limited sample size and inherent variations in training, match intensity, and player composition. Given the scarcity of workload monitoring and injury data sharing in the sports domain, this limitation is challenging to fully mitigate in practice.

Another important limitation is that our workload data was primarily derived from GPS‐based field sessions, while non‐locomotor training activities, including resistance and indoor exercises, were not consistently recorded due to equipment limitations. Although these sessions were relatively standardized across the team, their exclusion may have limited the completeness of overall workload representation in our models. Additionally, overuse injuries were not captured, as the study relied solely on routinely collected injury data. Future research could incorporate tools such as the Oslo Sports Trauma Research Center (OSTRC) Overuse Injury Questionnaire to better track these injuries. Furthermore, the limited data resources in this study prevented differentiation between training and match injuries or the identification of specific injury locations. One additional limitation is that although all non‐contact injuries, including those involving upper‐body regions, were included, the majority occurred in the lower extremities (73.5% in the present study). This may have biased the predictive value of GPS‐derived features, which primarily reflect locomotor activities such as running. Although cumulative running load and fatigue could also contribute indirectly to upper‐body injuries, the lack of detailed anatomical differentiation reduces the specificity of our findings. Such differentiation would provide valuable insights into context‐specific risk factors and help identify the most commonly injured areas, enabling the development of more targeted preventive strategies. Future research should address this limitation by incorporating more extensive data collection and analysis, improving the model's generalizability and practical applicability.

## 5 Practical Applications

The findings of this study provide practical guidance for workload management in rugby union during both training and matches. Injury risk factors vary significantly among players in different positions, enabling coaches and sports scientists to design more targeted training programs that align with specific positional demands. Training should focus on managing high‐intensity efforts and improving acceleration and deceleration mechanics to minimize injury risk. Additionally, improving endurance at moderate intensities to meet sustained tactical demands, as well as enhancing agility and speed control to reduce injuries caused by rapid changes in movement. Moreover, ACWR plays a critical role in influencing injury across various time windows. Therefore, carefully monitoring and managing ACWR during these key periods is essential for minimizing injury risk. Leveraging suitable ML models can enhance the precision of injury prediction, enabling teams to make more informed decisions.

## Conflicts of Interest

The authors declare no conflicts of interest.

## Supporting information


Supporting Information S1
