# Supplementary material for: Global Positioning System‐Derived Metrics and Machine Learning Models for Injury Prediction in Professional Rugby Union Players
Source: Eur J Sport Sci. 2025 Sep 24;25(10):e70057. doi: 10.1002/ejsc.70057 (PMC12460712; doi:10.1002/ejsc.70057)
Supplement: Supplementary file 1 — Supporting Information S1 [file EJSC-25-e70057-s001.docx]

# Classification ML algorithms

## Logistic Regression

Logistic Regression (LR) is a widely used parametric statistical model that estimates the probability of a binary event occurring based on one or more predictors (explanatory variables). Mathematically, this model first multiplies these predictors by their corresponding numerical parameters and then linearly combines them. The result of this combination is then fed into the logit function, which transforms the output of the linear combination into a probability value within the (0,1) range, thereby representing the nonlinear relationship between the dependent and explanatory variables. Due to its simplicity and effectiveness, LR has become one of the most commonly used models for handling binary classification problems (4).

LR equation:

$$y=\frac{e^{(b_{0}+b_{1}*x)}}{1+e^{(b_{0}+b_{1}*x)}}$$

Here, $y$ for predicted output, $b_{0}$ is the bias, and $b_{1}$ is the coefficient for the single input value $(x)$.

## Naïve Bayes

Naïve Bayes (NB) classifier is a simple probabilistic classifier based on Bayes' theorem, which classifies by calculating the probability that an instance belongs to each category. Its core assumption is that all features are mutually independent, meaning that the value of a feature given the class is independent of the values of other features. This "naïve" assumption greatly simplifies the computation. By applying Bayes' theorem, the classifier can predict the probability that an instance belongs to a particular category, considering the prior probability, likelihood, and normalization factor in its calculation.

The formula below is used to calculate the posterior probability of $C_{j}$ given data $d_{j}$ as follows:

$$P\boldsymbol{(}C_{j}\mid d_{j})=\frac{P\boldsymbol{(}d_{j}\mid C_{j})P\boldsymbol{(}C_{j})}{P\boldsymbol{(}d_{j})}$$

In this formula:

$P\boldsymbol{(}C_{j}\mid d_{j})$is the posterior probability of class $C_{j}$ given the data $d_{j}$, $P\boldsymbol{(}d_{j}\mid C_{j})$ is the likelihood of observing the data $d_{j}$ given class $C_{j}$, $P\boldsymbol{(}C_{j})$ is the prior probability of class $C_{j}$, $P\boldsymbol{(}d_{j})$ is the marginal probability of observing the data $d_{j}$, also known as the normalization factor (6).

## Support Vector Machine

Support Vector Machine (SVM), first proposed by Vapnik (3) within the framework of statistical learning theory, is a powerful tool for pattern recognition and classification. The core idea of SVM is to find an optimal hyperplane in an $n$-dimensional space that separates data points from different classes as effectively as possible. The distance between the hyperplane and the support vectors (i.e., the training data points closest to the separating boundary) is referred to as the margin. The goal of SVM is to maximize this margin, thereby enhancing the model's generalization capability and improving its performance on unseen data (1).

The basic condition for separating hyperplanes is given by:

$$y_{i}\left( \mathbf{w}^{\top}\mathbf{x}_{i}+b \right)\geq1, i=1,2,\ldots,n$$

Here, $\mathbf{w}^{\top}\mathbf{x}_{i}+b=0$ represents the hyperplane, and $y_{i}$ ensures that each sample is correctly classified with the required margin.

To handle the case where the dataset is not linearly separable, the slack variable $\xi_{i}$ is introduced to obtain the slack condition:

$$y_{i}\left( \mathbf{w}^{\top}\mathbf{x}_{i}+b \right)\geq1-\xi_{i}, \xi_{i}\geq0, i=1,2,\ldots,n$$

The goal is to minimize the following objective function to find the optimal hyperplane:

$$\min_{\mathbf{w},b,\boldsymbol{\xi}} \frac{1}{2}\|\mathbf{w}{\|}^{2}+C\sum_{i=1}^{n} \xi_{i}$$

$\frac{1}{2}\|\mathbf{w}{\|}^{2}$ represents the margin maximization (minimizing $\|\mathbf{w}\|$ ); $C\sum_{i=1}^{n} \xi_{i}$ penalizes misclassified samples, where $C>0$ controls the trade-off between the two.

By introducing Lagrange multipliers $\alpha_{i}\geq0$, the dual problem can be formulated as:

$$\max_{\alpha} \sum_{i=1}^{n} \alpha_{i}-\frac{1}{2}\sum_{i=1}^{n} \sum_{j=1}^{n} \alpha_{i}\alpha_{j}y_{i}y_{j}\mathbf{x}_{i}^{\top}\mathbf{x}_{j}$$

Subject to the constraints:

$$\sum_{i=1}^{n} \alpha_{i}y_{i}=0, 0\leq\alpha_{i}\leq C, i=1,2,\ldots,n$$

$\alpha_{i}$ represents the weight or contribution of the $i$-th sample to the decision function.

Support vectors are the samples where $\alpha_{i}>0$.

The decision function for classification is expressed as:

$$f(\mathbf{x})=sign\left( \sum_{i=1}^{n} \alpha_{i}y_{i}\mathbf{x}_{i}^{\top}\mathbf{x}+b \right)$$

If $f(\mathbf{x})>0$, the sample is classified as $+1$; if $f(\mathbf{x})<0$, it is classified as $-1$.

To handle nonlinear separability, the kernel function $K\left( \mathbf{x}_{i},\mathbf{x}_{j} \right)$ replaces the inner product $\mathbf{x}_{i}^{\top}\mathbf{x}_{j}$, resulting in the kernelized decision function:

$$f(\mathbf{x})=sign\left( \sum_{i=1}^{n} \alpha_{i}y_{i}K\left( \mathbf{x}_{i},\mathbf{x} \right)+b \right)$$

The kernel functions used in this study were:

Linear Kernel: $K\left( \mathbf{x}_{i},\mathbf{x}_{j} \right)=\mathbf{x}_{i}^{\top}\mathbf{x}_{j}$

Gaussian (RBF) Kernel: $K\left( \mathbf{x}_{i},\mathbf{x}_{j} \right)=exp\left( -\gamma\left\| \mathbf{x}_{i}-\mathbf{x}_{j} \right\|^{2} \right)$, where $\gamma>0$ controls the kernel width.

## Random Forest

Random Forest (RF) is an efficient parallel ensemble learning method. It uses bootstrap sampling to randomly draw multiple sub-training sets from the original training set and independently constructs a Decision Tree (DT) for each sub-training set. During the construction of each DT, RF also introduces randomness in feature selection. Specifically, at each node split, instead of choosing the best feature from all available features, a random subset of features is selected, and the best feature is chosen from this subset. Ultimately, RF aggregates the predictions of all DTs through majority voting to produce the final prediction.

The mathematical formula for RF classifier is:

$$n_{ij}=w_{l}C_{j}-w_{left(j)}C_{left(j)}-w_{right(j)}C_{right(j)}$$

$n_{ij}$ represents the importance of node $j$, $w_{l}$ is the weighted number of samples reaching node $j$, $C_{j}$ is the impurity value of node $j$, $left(j)$ denotes the child node from left split on node $j$, $right(j)$ denotes the child node from right split on node (5).

## eXtreme Gradient Boosting

eXtreme Gradient Boosting (XGBoost) model is an integrated learning algorithm that combines efficiency, flexibility, and accuracy. It is based on DTs as the base learners and is optimized using a gradient boosting framework. Compared to traditional methods, XGBoost significantly improves processing speed and efficiency, often by a factor of about ten. This improvement is due to its optimized distributed and parallelized algorithms and effective utilization of hardware resources. The algorithm enhances model performance by applying a second-order Taylor expansion to the loss function and introducing regularization terms in the objective function. This approach balances the reduction in the objective function with the model's complexity, effectively preventing overfitting while improving computational efficiency (2).

The objective function of XGBoost typically comprises two components: the training loss and a regularization term. These components can be expressed as follows:

$$Obj(\theta)=L(\theta)+\Omega(\theta)$$

Here, $L$ represents the training loss function, which evaluates the model's performance on the training dataset, while $\Omega$ denotes the regularization term designed to manage the model's complexity and mitigate overfitting.

The complexity of each tree is typically calculated using the following equation:

$$\Omega(f)=\gamma T+\frac{1}{2}\lambda\sum_{j=1}^{T} \omega_{j}^{2}$$

Here, $\Omega(f)$ represents the regularization term; $T$ refers to the total number of leaves; $\gamma$ is a parameter that controls the penalty for the number of leaves $T$ in the tree; $\omega$ represents the vector of leaf scores; $j$ represents the index of a leaf in the decision tree; $\lambda$ is a parameter that penalizes large leaf weights $\omega_{j}$.

The objective function of XGBoost, known as the structure score, is expressed as:

$$Obj=\sum_{j=1}^{T} \left[ G_{j}\omega_{j}+\frac{1}{2}\left( H_{j}+\lambda\right)\omega_{j}^{2} \right]+\gamma T$$

Where $\omega_{j}$ are independent of each other. The form $G_{j}\omega_{j}+\frac{1}{2}\left( H_{j}+\lambda\right)\omega_{j}^{2}$ is quadratic and the best $\omega_{j}$ for a given structure $q(x)$.

# REFERENCES

1. Burges CJ. A tutorial on support vector machines for pattern recognition. *Data mining and knowledge discovery* 2: 121-167, 1998.

2. Chen T, Guestrin C. Xgboost: A scalable tree boosting system. In: Proceedings of the Proceedings of the 22nd acm sigkdd international conference on knowledge discovery and data mining. 2016.

3. Cortes C. Support-Vector Networks. *Machine Learning*, 1995.

4. Kleinbaum DG, Klein M. Logistic regression. Statistics for biology and health. *Retrieved from DOI* 10: 978-971, 2010.

5. Shah K, Patel H, Sanghvi D, Shah M. A comparative analysis of logistic regression, random forest and KNN models for the text classification. *Augmented Human Research* 5: 12, 2020.

6. Van de Schoot R, Depaoli S, King R, et al. Bayesian statistics and modelling. *Nature Reviews Methods Primers* 1: 1, 2021.
